# Protein profiling of plasma proteins in dairy cows with subclinical hypocalcaemia

**DOI:** 10.1186/s13620-017-0082-0

**Published:** 2017-01-18

**Authors:** Ziling Fan, Shi Shu, Chuchu Xu, Xinhuan Xiao, Gang Wang, Yunlong Bai, Cheng Xia, Ling Wu, Hongyou Zhang, Chuang Xu, Wei Yang

**Affiliations:** Department of College of Animal Science and Veterinary Medicine, Heilongjiang Bayi Agricultural University, Daqing, 163319 China

**Keywords:** Dairy cows, iTRAQ, LC-MS/MS, Proteomics, Subclinical hypocalcaemia

## Abstract

Subclinical hypocalcaemia (SH) is an important metabolic disease in dairy cows that has a serious impact on production performance. The objective of this study was to investigate novel aspects of pathogenesis using proteomics technology to identify proteins that are differentially expressed in diseased and healthy animals. Dairy cows were divided into an SH group (T, *n* = 10) and a control group (C, *n* = 10) based on plasma calcium concentration. A total of 398 differentially expressed proteins were identified, of which 265 proteins were overlapped in the two parallel experiments. Of these, 24 differentially expressed proteins were statistically significant. Gene Ontology analysis yielded 74 annotations, including 7 cellular component, 55 biological process and 12 molecular function categories. Bioinformatics analysis indicated that calcium regulation, immune and inflammatory response, blood coagulation and complement pathway were all related to SH. Our iTRAQ/LC-MS/MS (isobaric tags for relative and absolute quantification/liquid chromatography-mass spectrometry/mass spectrometry) approach proved highly effective for plasma protein profiling of dairy cows with SH, and the results pave the way for further studies in this area.

## Findings

Animal husbandry, and dairy farming in particular, has grown considerably in China in recent years, and is now a major industry. However, the increase in dairy farming has been accompanied by the proliferation of metabolic diseases that have a significant deleterious effect on the production performance of dairy cows. Subclinical hypocalcaemia (SH) is one such metabolic disease that has no obvious clinical signs and can therefore be easily ignored. Biochemical tests show that the blood calcium (Ca) concentration of dairy cows with SH is between 1.38 and 2.00 mmol/L. This disease increases the risk of other perinatal diseases due to the reduced blood Ca concentration and muscle contractive capacity [1]. SH has been studied using proteomics approaches based on SELDI-TOF-MS (surface-enhanced laser desorption/ionization-time of flight-Mass spectrometry) [2, 3], 2D-DIGE (fluorescence two-dimensional differential gel electrophoresis) combined with MALDI-TOF-MS (matrix-assisted laser desorption/ionization-time of flight-mass spectrometry) [4] and metabolomics based on ^1^H-NMR (1hydrogen-nuclear magnetic resonance) [5]. However, these techniques have their limitation on studying differentially expressed proteins in dairy cows with SH. And our iTRAQ/LC-MS/MS technique can obtain more comprehensive information on differentially expressed proteins, which can further explore the pathogenesis and preventive methods of SH in dairy cows. In the present study, we used iTRAQ (isobaric tags for relative and absolute quantitation) to isotopically label and simultaneously analyze protein expression in diseased and healthy animals [6]. The objective of this study was to identify differentially expressed proteins in plasma from dairy cows with SH using iTRAQ/LC-MS/MS combined with bioinformatics analysis to provide novel insight into the pathogenesis of SH.

## Materials and methods

### Experimental animals

This experiment was conducted in strict accordance with the recommendations in the Guide for the Care and Use of Laboratory Animals of the National Institutes of Health located in United States. All experimental animals were treated according to the International Guiding Principles for Biomedical Research Involving Animals.

All cows were obtained from an intensive dairy farm located in Heilongjiang Province. All diets were in accordance with the Chinese standards for cattle breeding. All cows were fed a total mixed ration during the dry period, which consisted of 2.02 kg of concentrated feed, 17.37 kg of silage maize, and 4.2 kg of leymus chinensis, with a dry matter content of 42.9%. The net energy for dry cow of this diet was 1.3 Mcal/kg DM. The nutritional content was 12.89% crude protein, 2.83% fat, 49.21% neutral detergent fibre, 27.47% acid detergent fibre, and 71.71 g of Ca and 42.75 g of P per cow per day. The major mineral content was 91.81 mg of zinc, 59.29 mg of manganese, 16.60 mg copper, 0.44 mg of iodine, 0.25 mg of cobalt and 0.79 mg of selenium per cow per day. The dietary cation anion difference (DCAD) was 91 meq/kg DM. All cows were fed the same diet during the dry period.

Experimental Holstein dairy cows of a similar age, parity and body condition were selected. In total, 10 animals with SH were placed in T group, and 10 healthy cows were placed in C group. Cows were considered to be suffering from SH if within 1–3 days postpartum they displayed no obvious clinical signs but exhibited a plasma Ca concentration between 1.38 and 2.00 mmol/L. In addition, both groups contained only clinically normal cows in every other respect. In order to minimize individual differences between samples, 5 plasma samples from each group were mixed to generate one hybrid sample, and this was labelled using an iTRAQ kit and used in subsequent experiments. Basic information on dairy cows and experimental design are listed in Table 1.

**Table 1:** Information on experimental animals and iTRAQ labelling

	SH (T)		Control (C)	
	T1	T2	C1	C2
Age	3.2 ± 0.84	3.2 ± 0.84	2.8 ± 0.84	2.8 ± 0.84
Parity	5.2 ± 0.84	5.2 ± 0.84	4.8 ± 0.84	4.8 ± 0.84
BCS	3.25 ± 0.05	3.15 ± 0.06	3.20 ± 0.07	3.25 ± 0.06
Plasma Ca (mmol/L)	1.91 ± 0.08 ^A^	1.93 ± 0.08 ^A^	2.24 ± 0.08 ^B^	2.26 ± 0.09 ^B^
iTRAQ kit	114	115	116	117

*SH* subclinical hypocalcaemia, *BCS* body condition score, *Ca* calcium, *iTRAQ* isobaric tags for relative and absolute quantification. SH cows, *n* = 10; Control cows, *n* = 10; T1, T2, C1, C2, *n* = 5; mean ± standard deviation. The same capital letter in a row indicates no significant difference between groups (*P* > 0.05). Different capital letters in a row indicate highly significant differences between groups (*P* < 0.01)

### Collection of blood samples

All blood samples were obtained within 6 h of calving from tail veins of cows in the early morning, anticoagulated with heparin and immediately centrifuged at 3000 × *g* for 10 min at room temperature. The plasma were transferred to eppendorf tubes (1.5 mL) and stored at −80 °C until needed.

### Measurement of plasma Ca concentration

The plasma Ca concentration was measured using an automatic biochemical analyser (modular DPP, Roche Diagnostics GmbH) and a Ca plasma kit (651564-01, Roche Diagnostics GmbH).

### Elimination of high abundance proteins from plasma samples

In order to enrich low abundance proteins, high abundance proteins were eliminated according to the operating instructions supplied with the ProteoMiner kit (Bio-Rad, USA, catalog#s163-3007) following thawing and mixing of samples. Protein concentration was measured by the Bradford method to determine the loading quantity, and eliminated high abundance proteins were detected by SDS-PAGE (sodium dodecyl sulfate polyacrylamide gel electrophoresis).

### iTRAQ labelling

Plasma samples (100 μL) digested with protease were mixed with trypsase (dissolved in precooled deionized water) at a ratio of 20:1, digested again at 27 °C overnight, and centrifuged at 1000 × *g* for 30 s at 4 °C. iTRAQ reagent (in each tube) was dissolved completely in 70 μL of ethanol, and centrifuged at 1000 × *g* for 30 s at 4 °C. Treated plasma samples were labelled with treated iTRAQ reagent following standing for 2 h, and labelled plasma samples (1 μL) were vortexed and evaporated to dryness in vacuum. Information on iTRAQ labelling is shown in Table 1.

### Strong cation exchange (SCX) and high pH reverse fractionation

Peptides in dried plasma samples were dissolved in loading buffer (10 mmol/L K_3_PO_4_ pH 3.0, 25% ACN) (acetonitrile) and the pH was adjusted to 3. A PolyLC SCX chromatographic column (10 mm id × 14 mm, 12 μm, 300 A; PolyLC Inc, Columbia, MD, USA) was equilibrated in loading buffer, and 400 μg of sample was loaded onto the column and eluted with 2 ml loading buffer. Eluted peptides were evaporated to dryness in vacuum and separated by high pH reverse fractionation. Following drying in vacuum, peptides were dissolved in 150 μl of 2% ACN/0.5% FA (formaldehyde) and stored at −80 °C until needed for MS.

### Nano LC-MS/MS separation and MS identification

Samples were separated using an UltiMate 3000 RSLCnano nano-litre flow rate LC system (Thermo-Dionex, Sunnyvale, CA, USA), and MS was performed with a LTQ Orbitrap Velos (Thermo-Fisher Scientific, San Jose, CA, USA). Raw data were collected by Xcalibur 2.1 operation software (Thermo-Fisher Scientific), corrected using Proteome Discoverer 1.3 (Thermo) and exported as MGF formatted files. Mascot 2.3.02 (Matrix Science, Boston, MA, USA) was used for database searching, and processed data were used to search the NCBInr Bovine RefSeq database that contained 71,248 protein sequences when downloaded on April 11, 2011. The error rate of peptide and protein identification was <5%, and at least two unique peptides were identified for each protein. All potential SH-related proteins were subjected to quantitative analysis by Proteome Discoverer l.3 according to the pseudo-molecular- ion intensity obtained in the peptides report. The ratio of the quasi-molecular-ion intensity (114/116 and 115/117) was determined by each protein present in both T and C group.

### Statistical analysis

The age, parity, body condition score [7] and plasma Ca concentration of cows between groups were analyzed by one-way ANOVA using SPSS version 19.0 (IBM, Armonk, NY, USA). Differentially expressed proteins were analyzed by Venn analysis (VENN 2.1, http://bioinfogp.cnb.csic.es/tools/venny/) and Gene Ontology (GO) analysis (GOTERM_BP_ALL,GOTERM_CC_ALL, GOTERM _MF_ALL) using DAVID bioinformatics analysis software (v6.7, http://david.abcc.ncifcrf.gov/home.jsp).

## Results

### Information on experimental animals

The age, parity, body condition score and plasma Ca concentration of SH and control cows are listed in Table 1. The observed decrease in the plasma Ca concentration between T and C groups was the only significant difference.

### Venn analysis of experimental results

**Fig. 1:**
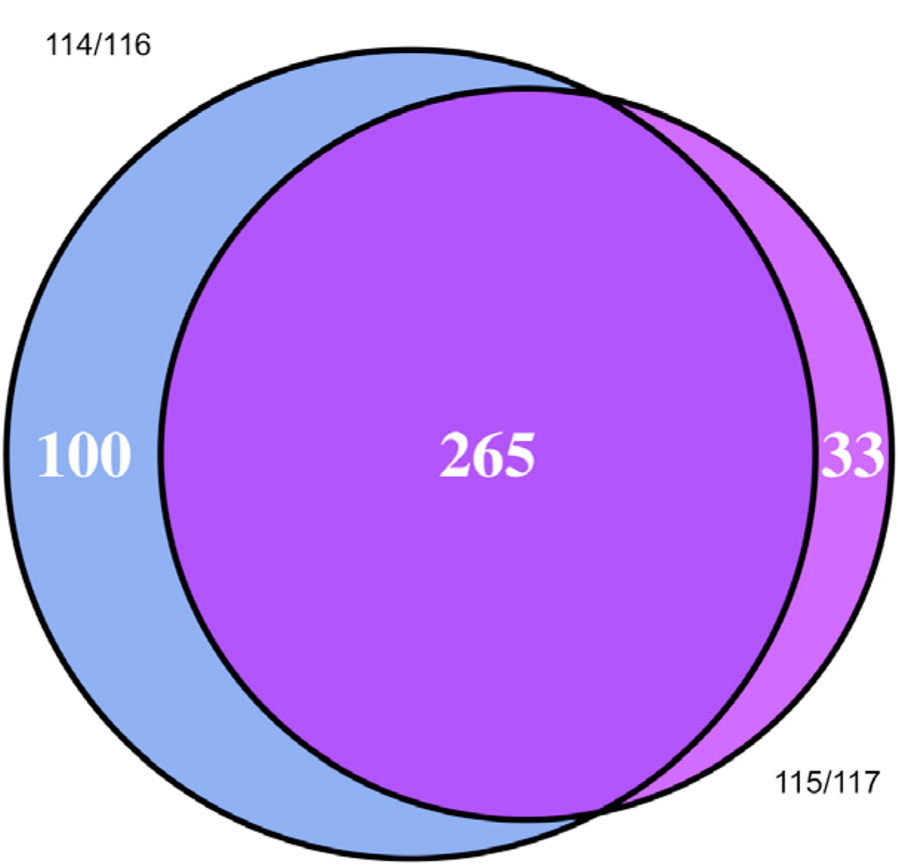
Venn analysis for 398 proteins differentially expressed in two parallel experiments (114/116 and 115/117). A total of 265 proteins are overlapping, and 100 and 33 proteins are unique to each parallel experiment

Data (114/116 and 115/117) were collected in two parallel experiments, and 365 and 298 proteins were identified in each parallel experiment, which resulted in 398 differentially expressed proteins. Venn analysis of all 398 differentially expressed proteins confirmed 265 overlapping proteins, and 100 and 33 unique proteins in each parallel experiment (Fig. 1). Overlapping proteins accounted for 66.58% of all identified proteins.

### Identification of differentially expressed proteins

Mean values of the fold change in expression were calculated for proteins overlapping (265) in the two parallel experiments. A mean value greater than 1.5 was considered up-regulated, and a mean value lower than 0.67 was considered down-regulated. As a result, 24 differentially expressed proteins were identified, comprising 21 that were up-regulated and 3 that were down-regulated (Tables 2 and 3).

**Table 2:** Proteins up-regulated among overlapping proteins

GI number	Protein name	Mean fold change
78369352	Complement component C9 precursor [Bos taurus]	1.55
94966809	Serum amyloid A-4 protein precursor [Bos taurus]	1.57
27807167	Peroxiredoxin-6 [Bos taurus]	1.60
358420568	PREDICTED: complement C4-A-like [Bos taurus]	1.62
155372183	Carboxypeptidase N subunit 2 precursor [Bos taurus]	1.62
83035071	Fermitin family homolog 3 [Bos taurus]	1.66
77735579	Fibrinogen-like protein 1 precursor [Bos taurus]	1.66
27806297	Flavin reductase (NADPH) [Bos taurus]	1.66
155372051	Tropomyosin alpha-4 chain [Bos taurus]	1.68
115497210	Complement C1s subcomponent precursor [Bos taurus]	1.71
164450479	Kininogen-2 isoform I precursor [Bos taurus]	1.73
154707858	Cadherin-17 precursor [Bos taurus]	1.75
61888874	Transgelin-2 [Bos taurus]	1.79
78042516	Phospholipid transfer protein precursor [Bos taurus]	1.81
221136893	C-reactive protein precursor [Bos taurus]	1.90
95147666	Periostin precursor [Bos taurus]	1.93
114051505	Serpin H1 precursor [Bos taurus]	2.02
84579853	Lipopolysaccharide-binding protein precursor [Bos taurus]	2.34
41386760	Monocyte differentiation antigen CD14 precursor [Bos taurus]	2.59
115497340	Serum amyloid A protein precursor [Bos taurus]	4.54
94966763	Haptoglobin precursor [Bos taurus]	8.65

GI (genInfo identifier). Proteins with a mean value greater than 1.5 are considered up-regulated, while those with a mean value lower than 0.67 are considered down-regulated

**Table 3:** Proteins down-regulated among overlapping proteins

GI number	Protein name	Mean fold change
155371855	Platelet factor 4 precursor [Bos taurus]	0.48
45429979	Spleen trypsin inhibitor I precursor [Bos taurus]	0.54
41386685	Thrombospondin-1 precursor [Bos taurus]	0.63

GI (genInfo identifier). Proteins with a mean value greater than 1.5 are considered up-regulated, while those with a mean value lower than 0.67 are considered down-regulated

### Bioinformatics analysis of differentially expressed proteins

**Fig. 2:**
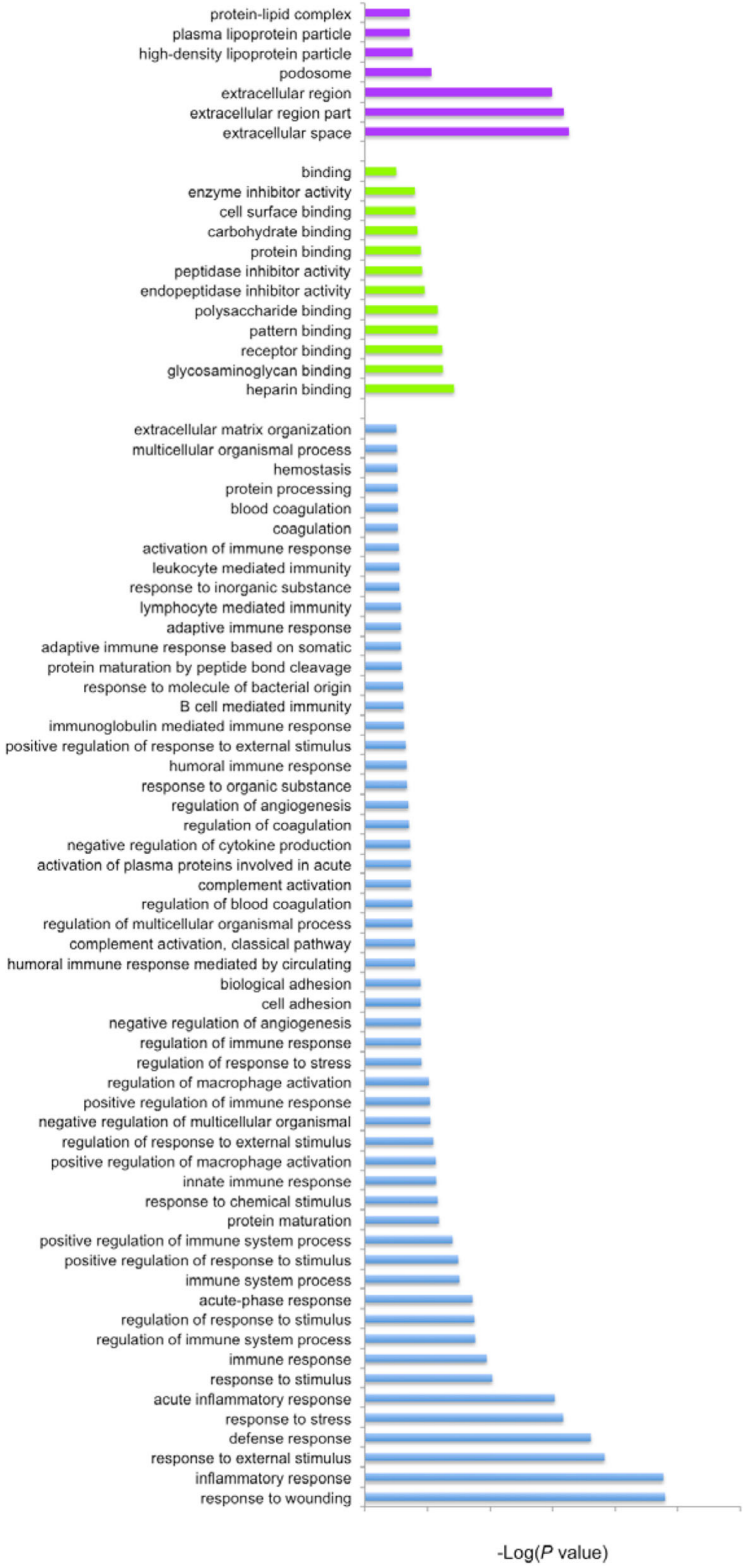
Gene Ontology analysis for 24 differentially expressed proteins. Purple bars indicate cellular component, blue bars represent biological process, and green bars correlate with molecular function

All 24 differentially expressed proteins were subjected to function enrichment analysis of cell component, molecular function and biological process categories (Fig. 2). In total, 74 annotations were obtained, comprising 7 cell components, 12 molecular functions and 55 biological processes.

GO analysis revealed that the most highly represented biological process terms were ‘inflammatory reaction, immunoreaction and resistance function’, while the top molecular function terms were ‘cell’ and ‘molecular’, and the dominant cell component terms were ‘extracellular domain’ and ‘lipoprotein’.

## Discussion

In this study, iTRAQ and LC-MS/MS were used for plasma protein profiling of SH in dairy cows. A total of 398 differentially expressed proteins were identified, of which 265 proteins overlapped in the two parallel experiments. Of these, 24 differentially expressed proteins were statistically significant, of which 21 were up-regulated and 3 were down-regulated. Based on bioinformatics analysis, most differentially expressed proteins were associated with inflammatory action and immunological processes.

### Differentially expressed proteins related to blood Ca concentration

Two of the up-regulated proteins were identified as potentially related to blood Ca concentration, namely cadherin and periostin. Cadherin is a single chain transmembrane glycoprotein involved in cell adhesion, forming adherens junctions and binding cells together within tissues, and its activity is Ca dependent. Cadherin plays an important role in the formation of osteoblasts and bone by controlling adhesion and interaction between cells [6, 8, 9], and it has also been linked to cell recognition and sorting, boundary formation and maintenance, coordination of cell motility, and maintenance of cell structure [10]. Meanwhile, the 811 amino acid periostin induces cell adhesion and separation, and members of the periostin family are characterized by a cysteine-rich N-terminal region [11]. Studies have indicated that periostin is a novel member of the vitamin K-dependent gamma-carboxylate protein family [12]. Periostin is involved in both normal physiological and pathological processes, including vascular disease [13], trauma repair [14], bone formation [15] and formation of tumours [16]. Our GO analysis results indicated that cadherin and periosteal cells function in cell adhesion and protein binding.

In general, clinical hypocalcaemia (CH) is diagnosed based on clinical signs and blood Ca concentration. However, accurate diagnosis of SH in dairy cows, which has been linked to various diseases affecting productivity in transition dairy cows, depends on the measurement of blood calcium. Up-regulation of the Ca-associated cadherin and periostin in SH suggests these proteins may initiate a mechanism that stimulates blood Ca when levels are decreased. The exact mechanism through which these proteins operate in dairy cows with SH requires further study.

### Differentially expressed proteins related to immune response and inflammatory reaction

SH is a well-characterised metabolic disease which has the high prevalence in the first week of lactation in dairy cows [17] and the high incidence of peripartum problems in dairy cows. Furthermore, immune cells in cows with SH display impaired function [18]. Our bioinformatics analysis indicated that most proteins differentially expressed in diseased animals were related to immune response and inflammatory reaction. These included serum amyloid A protein and haptoglobin. Haptoglobin is an acidoglycoprotein that is highly expressed in blood and is believed to act as a cytokine following release from activated macrophages during the initial stages of pathogenic infection and tissue damage, which can stimulate the liver to produce acute phase reactive protein. Expression of haptoglobin in the blood is increased during stress or following pathogen-induced damage [19].

Serum amyloid A is mainly synthesized by the liver and can exist in acute phase amyloid A and structural amyloid protein forms. Expression of serum amyloid A can be up-regulated 100-fold within 1–2 days of an organism suffering from physical damage, stress or microbial infection [20]. As mentioned above, dairy cows with SH show no obvious clinical signs. However, our results indicated that diseased animals do show hypoimmunity. Dairy cows with metabolic diseases such as SH are therefore more likely to contract other more debilitating infectious and non-infectious diseases that could greatly influence production performance.

### Differentially expressed proteins related to blood coagulation and complement pathways

Previous studies on CH and SH in dairy cows reported that the occurrence of CH is related to blood coagulation and complement pathways [3, 21–23], and our results are consistent with this observation. A number of differentially expressed proteins identified in the present study were related to blood coagulation and complement pathways, specifically platelet factor, serpin and complement factor. Complement and coagulation pathway proteins such as serpins and complement factors are known to be highly dependent on the plasma Ca concentration [24, 25]. Serpins play an important role in coagulation, fibrinolysis and the protection of neurons, while platelet factors assist adhesion and aggregation of platelets, and accelerate blood coagulation. Many complement factors also stimulate the production of specific antibodies and therefore boost the immune system. Complement activation ultimately results in the formation of membrane attack complexes that attack and kill pathogenic microorganisms [26]. Ca is an important factor in blood coagulation, and malfunctions can occur if the blood Ca concentration is reduced. In this study, we identified a number of differentially expressed proteins involved in coagulation and complement pathways. We speculate that reduced blood Ca in dairy cows with SH may alter key metabolic and immunological pathways, and lead to diminished production performance and hypoimmunity. These results therefore indicate that CH and SH could serve as recognizable risk factors for diseases affecting productivity in dairy cows such as mastitis, ketosis, retained placenta, displaced abomasum and uterine prolapse [27].

## Conclusion

In this study, we identified proteins that were differentially expressed in dairy cows with SH through a combined proteomics and bioinformatics approach. A total of 398 differentially expressed proteins were obtained, of which 24 were statistically significant and confirmed by MS. These proteins were mainly associated with blood Ca concentration, coagulation and complement pathways. Our results provide novel insight into SH in dairy cows.

## Abbreviations

ACN: Acetonitrile
Ca: Calcium
CH: Clinical hypocalcaemia
DCAD: Dietary cation anion difference
FA: Formaldehyde
SCX: Strong cation exchange
SH: Subclinical hypocalcaemia
